# TGF-β1-induced RAP2 regulates invasion in pancreatic cancer

**DOI:** 10.3724/abbs.2022015

**Published:** 2022-02-25

**Authors:** Kaizhou Jin, Chen Liu, He Cheng, Qinglin Fei, Qiuyi Huang, Zhiwen Xiao, Xianjun Yu, Weiding Wu

**Affiliations:** 1 Department of Pancreatic Surgery Fudan University Shanghai Cancer Center Shanghai 200032 China; 2 Department of Oncology Shanghai Medical College Fudan University Shanghai 200032 China; 3 Shanghai Pancreatic Cancer Institute Shanghai 200032 China; 4 Pancreatic Cancer Institute Fudan University Shanghai 200032 China

**Keywords:** pancreatic cancer, RAP2, TGF-β1, c-Myc, invasion

## Abstract

Pancreatic cancer is highly lethal due to its aggressive invasive properties and capacity for metastatic dissemination. Additional therapeutic targets and effective treatment options for patients with tumours of high invasive capacity are required. Ras-related protein-2a (RAP2) is a member of the GTP-binding proteins. RAP2 has been reported to be widely upregulated in many types of cancers via regulating cytoskeleton reorganization, cell proliferation, migration, and adhesion, as well as inflammation. As a member of the RAS oncogene family, which has been demonstrated to drive pancreatic cancer oncogenesis and many other malignancies, the physiological roles of RAP2 in pancreatic cancer have seldom been discussed. In the present study, we explored the correlation between RAP2 expression and the prediction of overall survival of pancreatic cancer patients. Mechanistic studies were carried out to shed light on the role of RAP2 in pancreatic cancer invasion and how RAP2 is regulated in the invasive process. Our results demonstrated that patients with higher RAP2 expression showed unfavourable prognoses. *In vitro* studies demonstrated that silencing of *RAP2* inhibited the invasion of pancreatic cancer cells. Moreover, our results demonstrated that transforming growth factor-β1 (TGF-β1), an inducer of the metastatic potential of pancreatic cancer cells, regulates the expression of RAP2 via the transcription factor c-Myc. In conclusion, the present study uncovered RAP2 as a novel predictive marker and therapeutic target for pancreatic cancer.

## Introduction

Pancreatic cancer is a highly lethal disease, and its mortality rate is almost equal to the incidence of the disease [1]. Despite recent advances in the diagnosis and treatment strategies for the cancer, the 5-year survival rate has remained at approximately 6% for many years, and pancreatic cancer still remains one of the leading causes of cancer-related deaths worldwide [ 2, 3]. Most pancreatic cancer patients die of invasion and metastasis to the regional lymph nodes and distant organs [4]. Therefore, there is an urgent need for identifying predictive markers to assist the regulation of the invasion of pancreatic cancer.

RAP proteins belong to the RAS family of small GTPases [5]. These proteins constitute two subfamilies including RAP1 and RAP2, and each family contains two isoforms (A and B) displaying 95% and 90% identity for RAP1 and RAP2, respectively [5]. Similar to other Ras-related GTPases, RAP proteins function as binary switches by cycling between two interconvertible states: a GDP-bound inactive and a GTP-bound active form [6]. RAP protein members play vital roles in the ability of cells to respond to environmental changes [7]. RAP can regulate cell polarity and actin-based morphology in yeast, *Drosophila* and *Xenopus embryos* [8]. In mammalian cells, RAP could regulate cytoskeleton dynamics and play important roles in cell/matrix and cell/cell adhesion through the effects of integrin activation and cadherin-mediated cell/cell adhesion [9]. Alterations in the cytoskeleton, cell/cell adhesion and integrin activation are critical nodes in the invasiveness and metastasis of carcinomas [ 10, 11]. The functions of RAP1 have been widely studied in many types of cancers and it plays diverse roles depending on the cancer type [ 12, 13]. As an important protein that shares many similarities with RAP1, the functions of RAP2 have seldom been reported in cancer, especially pancreatic cancer. In colorectal cancer, the RAP2 isoform RAP2C has been reported to weaken the migration and invasion of colorectal cancer cells via suppressing the epithelial mesenchymal transition (EMT) [14]. In prostate cancer cells, RAP2 can regulate androgen sensitivity and suppress androgen-stimulated growth [15]. Previous studies demonstrated that RAP2 is essential for the Wnt/β-catenin pathway during the body axis specification in *Xenopus embryos* [16]. Mechanistic studies demonstrated that RAP2 could physically interact with lipoprotein receptor-related protein 6 (LRP6) and stabilize LRP6 protein to regulate Wnt/β-catenin signalling [17]. Constitutive activation of Wnt/β-catenin signalling is a driving force for the oncogenesis and metastasis of pancreatic cancer [18]. Therefore, we assumed that RAP2 might play tumour-promoting roles in pancreatic cancer.

## Material and Methods

### Cell culture

The human pancreatic cancer cell lines PANC-1 and SW1990 were obtained from American Type Culture Collection (ATCC, Manassas, USA). The cells were cultured according to the standard protocols that were provided by the supplier. In brief, the PANC-1 cells were cultured in Dulbecco’s Modified Eagle’s Medium (DMEM; Invitrogen, Carlsbad, USA), containing foetal bovine serum (FBS; Invitrogen) at a final concentration of 10%. SW1990 cells were maintained in ATCC-formulated Leibovitz’s L-15 medium (ATCC) supplemented with FBS at a final concentration of 10%. Pancreatic cancer-associated fibroblasts (CAFs) were kindly provided by Dr. Chao Qu from Shanghai Cancer Center (Shanghai, China).

### Plasmids

To silence RAP2 expression, pLKO.1 TRC cloning vector (Plasmid 10878; Addgene, Watertown, USA) was used and the sequence of 21 bp target against RAP2 was 5′-CGGCACCTTCATCGAGAAATA-3′, and Scramble shRNA (plasmid 1864; Addgene) was used as the control vector. The sequence of 21 bp target against c-Myc was 5′-CCTGAGACAGATCAGCAACAA-3′. The promoter region of *RAP2* from −2000 to +200 was amplified from the genomic DNA of the PANC-1 cells and ligated into Promega’s pGL3-Basic vector (Promega, Madison, USA) to generate the pGL3-RAP2 construct. The mutated *RAP2* promoter luciferase construct was generated by using TOYOBO’s KOD-Plus Mutagenesis Kit (Toyobo, Osaka, Japan).

### RNA isolation and quantitative real-time PCR

Total RNA was obtained using TRIzol reagent (Invitrogen), and cDNA was reverse transcribed by reverse transcription with the PrimeScript RT reagent Kit (TaKaRa, Dalian, China). Gene expression status and control β-actin gene were determined by quantitative real-time PCR. All reactions were run in triplicate. Primer sequences are listed in Table 1. Relative quantification was then performed using the ∆∆Ct method with *β-actin* as the reference gene.

**Table:** **Table1** Sequences of primers used in this study

Name	Primer sequence
*RAP2* forward	5′-ACATCAAGCCCATGCGGGACCAGA-3′
*RAP2* reverse	5′-GTCAGGCTGAGCAGCATAGTTCAT-3′
*c-Myc* forward	5′-TCCACTCGGAAGGACTATCCTGCT-3′
*c-Myc* reverse	5′-AGCTCCGTTTTAGCTCGTTCCTC-3′
*β-actin* forward	5′-ACCACCATGTACCCTGGCATTGCC-3′
*β-actin* reverse	5′-GAAGCATTTGCGGTGGACGATGG-3′

### Protein extraction and western blot analysis

PANC-1 and SW1990 were washed twice with PBS and lysed for 10 min in RIPA buffer (20 mM Tris, pH 8.0, 150 mM NaCl, 1% NP40, 10% glycerol and 20 μM EDTA) that was supplemented with protease and phosphatase inhibitors. Cell debris was removed by centrifugation at 10,000 *g* for 10 min at 4°C. Then, 20 μg of whole cell lysates were denatured in SDS loading buffer and subjected to electrophoresis in a denaturing 10% SDS-polyacrylamide gel. The samples were then transferred to a membrane for subsequent blotting with specific antibodies, including anti-RAP2 antibody (Sigma-Aldrich, St Louis, USA), anti-N-cadherin antibody (Cell Signaling Technology, Beverly, USA), anti-Vimentin antibody (Cell Signaling Technology), anti-E-cadherin antibody (Cell Signaling Technology), anti-c-Myc and anti-β-actin antibodies (Proteintech, Rosemont, USA), followed by incubation with the corresponding horseradish peroxidase (HRP)-linked anti-mouse or rabbit IgG secondary antibodies (Cell Signaling Technology). SuperSignal West Pico PLUS (Invitrogen) was utilized as HRP substrate to visualize the protein bands. Target proteins were detected using the Fujifilm LAS-3000 imaging system.

### Transwell invasion assay

A 24-well Transwell chamber with an 8-μm-pore PET membrane (BD Biosciences, San Diego, USA) was used to conduct invasion assays. The lower chamber was filled with 800 μL media containing 10% FBS. Subsequently, approximately 6 × 10 ^4^ PANC-1 or SW1990 cells were seeded in 200 μL medium without serum in the top chamber for the migration assay, and 10 × 10 ^4^ cells were seeded into the top chamber with a Matrigel-coated membrane (BD) for the invasion assays. The cells were allowed to migrate at 37°C for 24 h with 5% CO _2_. After removal of the non-migrated or non-invaded cells, the remaining cells were washed, fixed, and stained with crystal violet. The number of migrating and invading cells was counted in six randomly selected fields at 100× magnification. Experiments were performed at least in triplicate.

### Dual-luciferase assay

To examine the impact of c-Myc on RAP2 expression, the pGL3-RAP2 vector was co-transfected with the c-Myc and Renilla luciferase vectors into PANC-1 and SW1990 cells. Luciferase activity was measured by using dual-luciferase assay kit (Beyotime Biotechno-logy, Shanghai, China) according to the manufacturers’ protocol.

### Chromatin immunoprecipitation assay

To test whether c-Myc could bind to the *RAP2* promoter, chromatin immunoprecipitation (ChIP) assay was performed by using EZ ChIP kit (Merck-Millipore, Billerica, USA) according to the manufacturers’ recommendations. ChIP grade anti-c-Myc antibody was obtained from Abcam (Cambridge, UK). The primer sequences to detect the c-Myc-bound *RAP2* promoter were: forward 5′-TCACCTACGATTGGTTGGCAGAG-3′ and reverse 5′-AACAGCCAGCTAGGGGCATCTAGT-3′.

### Tissue specimens and immunohistochemistry assay

The clinical tissue samples used in the present study were histopathologically and clinically diagnosed as pancreatic cancer at Fudan University Shanghai Cancer Center (FUSCC). Prior patient consent and approval were obtained from the Institutional Research Ethics Committee of FUSCC. Immunohistochemistry (IHC) staining was carried out according to a previous report [19]. The anti-RAP2 antibody used for IHC staining was purchased from Abcam.

### Statistical analysis

Statistical analysis was performed by using SPSS software (Version 17.0, IBM Corp) using Independent *t*-test (two tailed) or two-way ANOVA. Survivals were evaluated using the Kaplan–Meier method, and significance was measured using the log-rank test. Statistical significance was based on two-sided *P* values of <0.05.

## Results

### RAP2 predicts an unfavourable prognosis of pancreatic cancer

**Figure 1:**
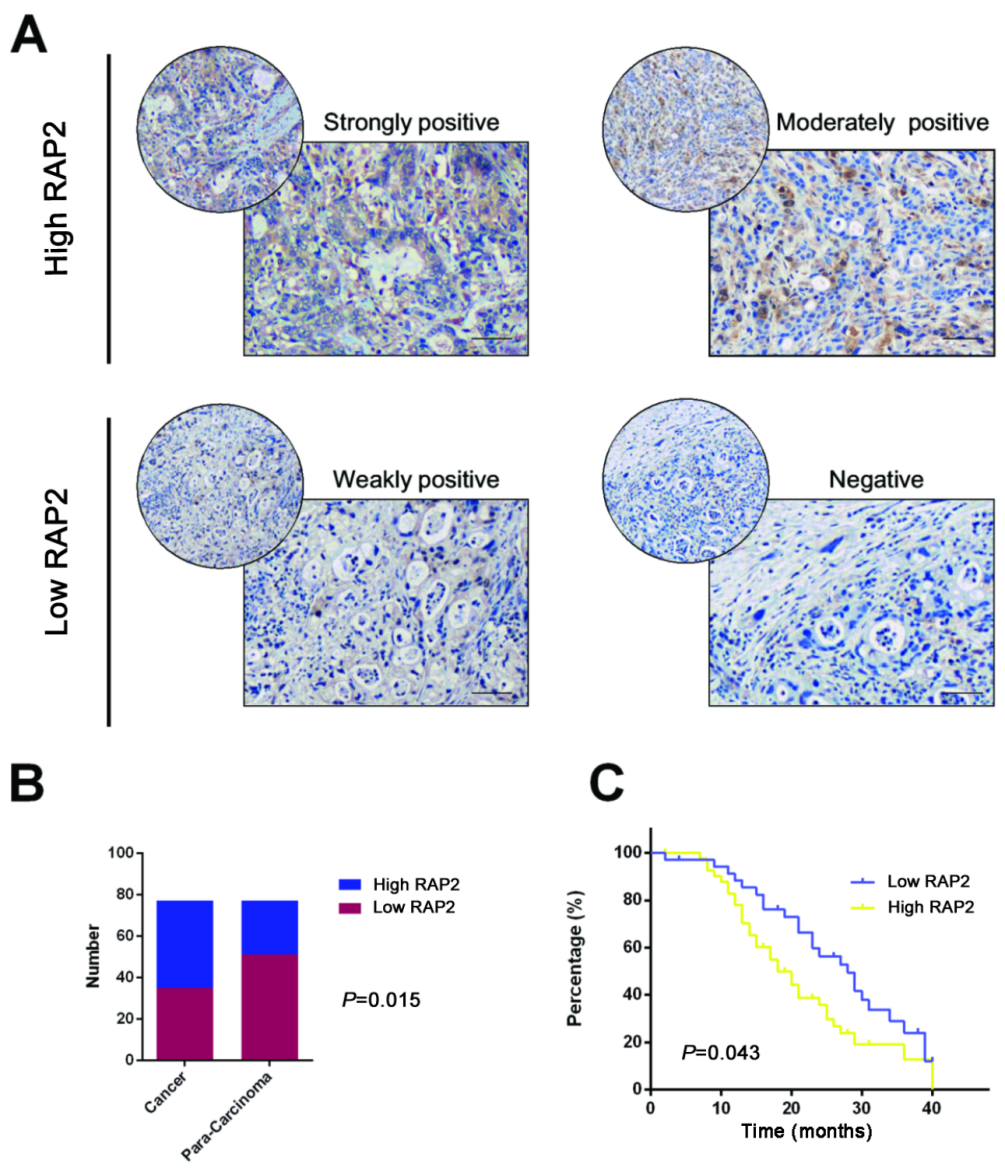
Figure1 RAP2 predicts unfavorable prognosis of pancreatic cancer (A) Immunohistochemical analysis for the tissue RAP2 expression in the FUSCC cohort at high magnification (400×). Scale bar=50 μm (black line at the bottom right). (B) The expression of RAP2 in pancreatic cancer and para-carcinoma tissue. (C) High level of tissue RAP2 expression predicts a poor prognosis to pancreatic cancer in the FUSCC cohort.

To evaluate the clinical significance of RAP2 in pancreatic cancer, we examined the expression status of RAP2 by IHC staining. The tissue samples were divided into High RAP2 (n=34) and Low RAP2 (n=43) based on their IHC staining intensities for RAP2 ( Figure 1A). Subsequent analysis demonstrated that RAP2 displayed a higher expression pattern in tissues from pancreatic cancer patients ( Figure 1B). Moreover, patients with higher RAP2 expression displayed worse outcomes ( Figure 1C). The clinicopathological features are listed in Supplementary Table S1.

### Silencing of *RAP2* inhibits the invasiveness of pancreatic cancer cells

**Figure 2:**
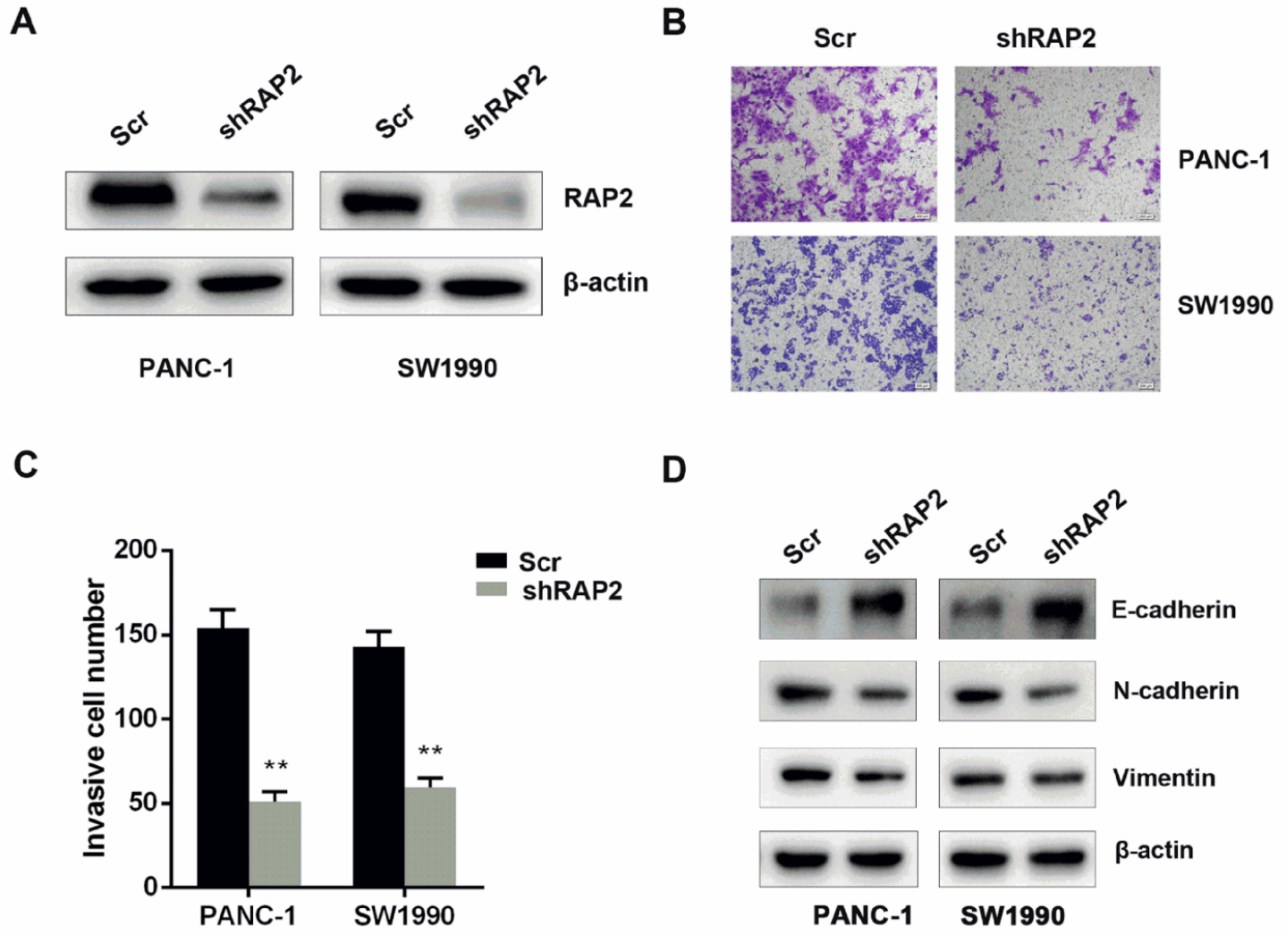
Figure2 Silencing of *RAP2* inhibits the invasiveness of pancreatic cancer cells (A) RAP2 was silenced in PANC-1 and SW1990 cells. (B) Silencing of RAP2 deceased the invasion of PANC-1 and SW1990 cells (magnification, 100×). (C) The relative number of migrated cells was calculated and shown as the mean±SD. (D) Knockdown of RAP2 could attenuate the expressions of N-cadherin and Vimentin, while the level of E-cadherin was significantly increased when RAP2 was knockdown. **P<0.01.

To validate the impact of RAP2 on migration and invasion in pancreatic cancer cells, RAP2 was silenced in PANC-1 and SW1990 cells ( Figure 2A). Next, we performed Transwell invasion assays to examine the role of RAP2 in the migration of pancreatic cells. Silencing of *RAP2* inhibited the invasive capacity of PANC-1 and SW1990 cells ( Figure 2B,C). In addition, the impact of RAP2 on the expression status of invasion and metastasis markers including E-cadherin, N-cadherin and Vimentin was examined by western blot analysis. In PANC-1 and SW1990 cells, knockdown of RAP2 attenuated the expressions of N-cadherin and Vimentin, while the level of E-cadherin was increased significantly ( Figure 2D).

### RAP2 is a downstream target of c-Myc in pancreatic cancer

**Figure 3:**
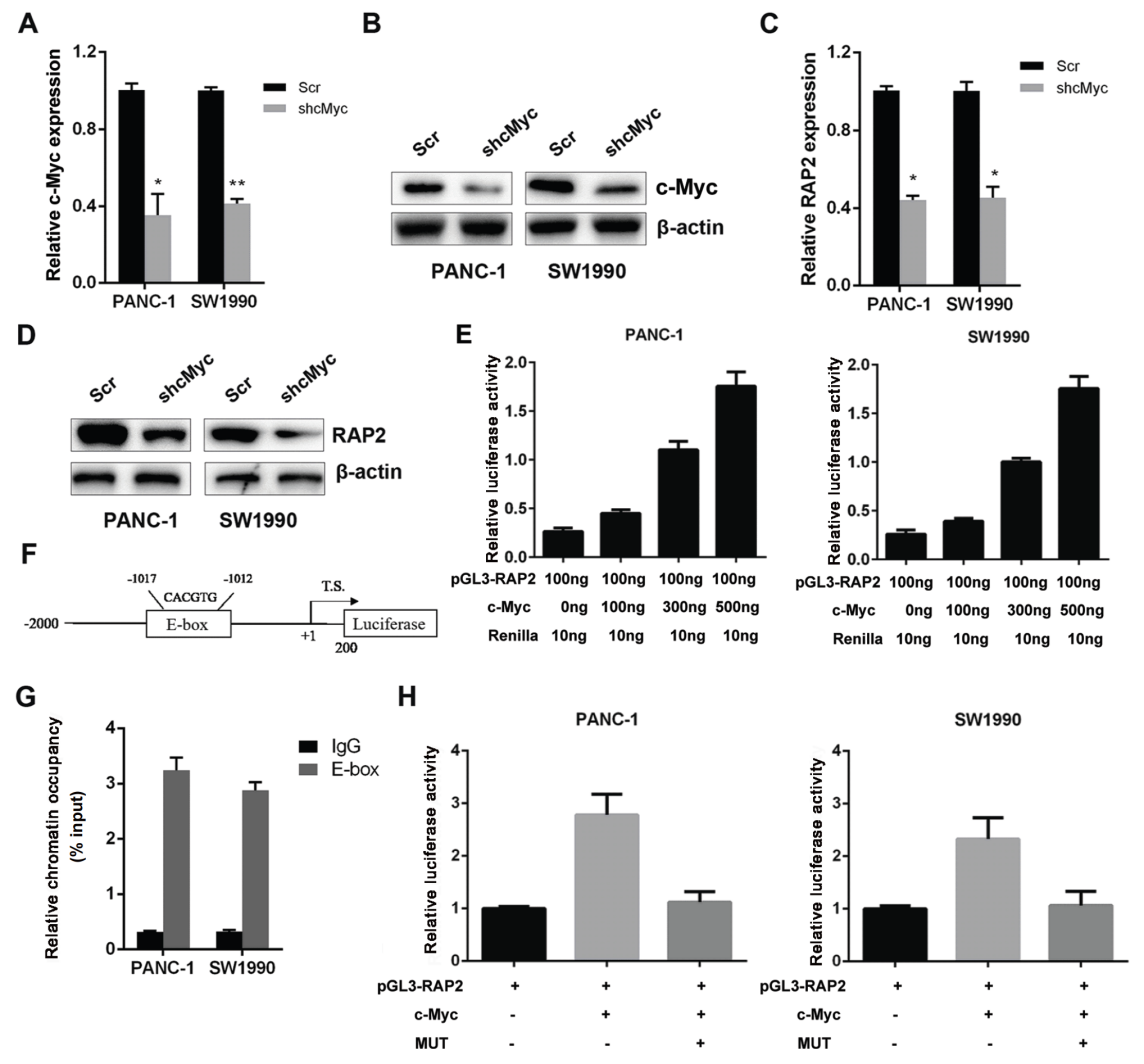
Figure3 RAP2 is a downstream target of c-Myc in pancreatic cancer (A) The mRNA expression level of c-Myc was decreased in PANC-1 and SW1990 cells. (B) The protein expression level of c-Myc was decreased in PANC-1 and SW1990 cells. (C) Decrease of RAP2 at mRNA level in c-Myc-silenced PANC-1 and SW1990 cells. (D) Decrease of RAP2 at protein level in c-Myc-silenced PANC-1 and SW1990 cells. (E) c-Myc increased RAP2 promoter activity in a dose-dependent manner in PANC-1 and SW1990 cells. (F) The position of the c-Myc–binding region. (G) c-Myc bound to the putative E-box on RAP2 promoter in PANC-1 and SW1990 cells. (H) c-Myc cannot regulate the luciferase activity of the mutant construct. *P<0.05,**P<0.01.

c-Myc is a master regulator of oncogenesis and progression in cancer cells [20]; thus, we asked whether it could regulate the expression of RAP2 in pancreatic cancer cells. First, we silenced the expression of c-Myc in PANC-1 and SW1990 cells and confirmed the silencing efficacy by quantitative PCR and western blot analysis ( Figure 3A,B). In c-Myc-silenced PANC-1 and SW1990 cells, we observed a decrease of RAP2 at both the mRNA and protein levels ( Figure 3C,D). This result suggested that RAP2 might be a downstream target of c-Myc. To prove this hypothesis, we cloned the *RAP2* promoter region from −2000 to +200 into the pGL3-Basic vector. Subsequent promoter luciferase assays demonstrated that c-Myc increased *RAP2* promoter activity in a dose-dependent manner in PANC-1 and SW1990 cells ( Figure 3E). In the promoter region, we identified a putative c-Myc binding E-box region composed of CACGTG from −1017 to −1012 ( Figure 3F). The ChIP assay results demonstrated that c-Myc could bind to the putative E-box in the *RAP2* promoter in PANC-1 and SW1990 cells ( Figure 3G). Then, we mutated the E-box CACGTG into TACATG, and the dual luciferase results demonstrated that in response, c-Myc was unable to regulate the mutated construct in the luciferase activity ( Figure 3H). Therefore, RAP2 is a transcriptional target of c-Myc in pancreatic cancer cells.

### RAP2 expression is responsible for TGF-β1/c-Myc axis-induced invasiveness of pancreatic cancer cells

**Figure 4:**
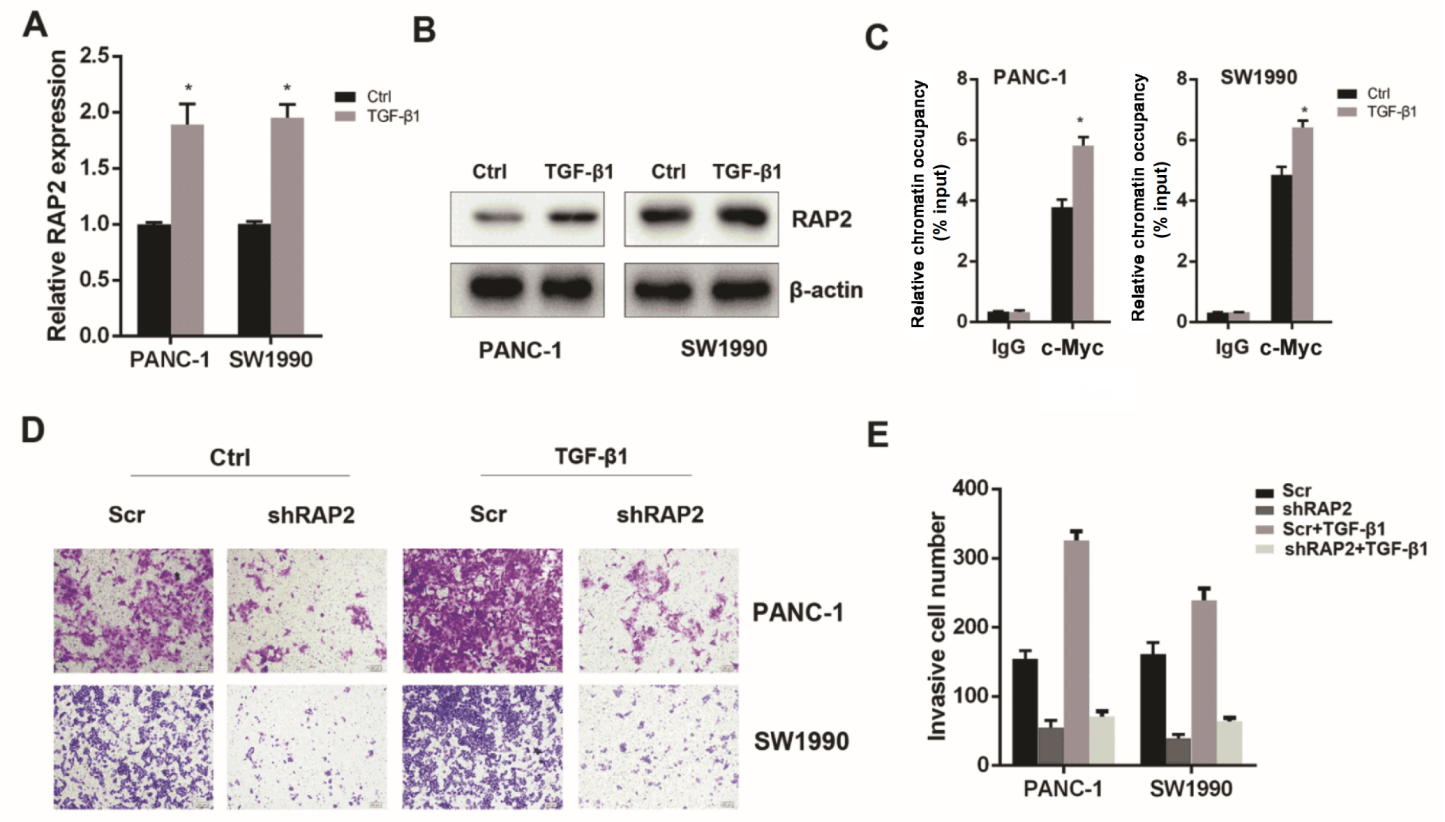
Figure4 RAP2 expression is responsible for TGF-β1/c-Myc axis-induced invasiveness of pancreatic cancer cells (A) TGF-β1 treatment (10 ng/mL, 48 h) increased the expression of RAP2 at mRNA level. (B) TGF-β1 increased the expression of RAP2 at protein level. (C) TGF-β1 treatment increased the occupancy status of c-Myc on the RAP2 promoter region. (D) TGF-β1 treatment induced migration and invasion capacity of PANC-1 and SW1990 cells (magnification, 100×). (E) Silencing of RAP2 in PANC-1 and SW1990 cells mitigated the increase in invasiveness conferred by TGF-β1.*P<0.05.

TGF-β1 is associated with receptor kinases that mediate phosphorylation-dependent signalling to downstream mediators, mainly the SMAD proteins [21]. Numerous studies have reported that TGF-β1 induces the activation and phosphorylation of SMAD2 and SMAD3, leading to their nuclear translocation and subsequent induction of c-Myc expression. Moreover, TGF-β1 induces ERK activation, which is a driving force for c-Myc post-translational stabilization. Therefore, TGF-β1 can induce the activation of c-Myc and the resultant invasiveness of cancer cells [ 22– 25]. Thus, we asked whether RAP2 is a downstream target of the TGF-β1/c-Myc axis and whether RAP2 is responsible for the TGF-β1/c-Myc-induced invasiveness. First, we treated the PANC-1 and SW1990 cells with 10 ng/mL TGF-β1 for 48 h [26] and assessed the impact of TGF-β1 on RAP2 expression. Real-time PCR and western blot analysis results demonstrated that TGF-β1 increased the expression of RAP2 at both the mRNA and protein levels ( Figure 4A,B). Next, we performed ChIP assays to observe the occupancy status of c-Myc in the *RAP2* promoter. Quantitative ChIP further confirmed that TGF-β1 treatment increased the occupancy status of c-Myc in the *RAP2* promoter region ( Figure 4C). Ultimately, we confirmed the impact of RAP2 on TGF-β1/c-Myc-induced invasiveness of pancreatic cancer cells. Transwell invasion assay results demonstrated that silencing of *RAP2* in PANC-1 and SW1990 cells could mitigate the increase in their invasiveness conferred by TGF-β1 ( Figure 4D,E). Therefore, TGF-β1/c-Myc induces the increase in RAP2, which is responsible for the TGF-β1/c-Myc-induced invasiveness of pancreatic cancer cells.

### CAFs induce RAP2 expression in pancreatic cancer cells

**Figure 5:**
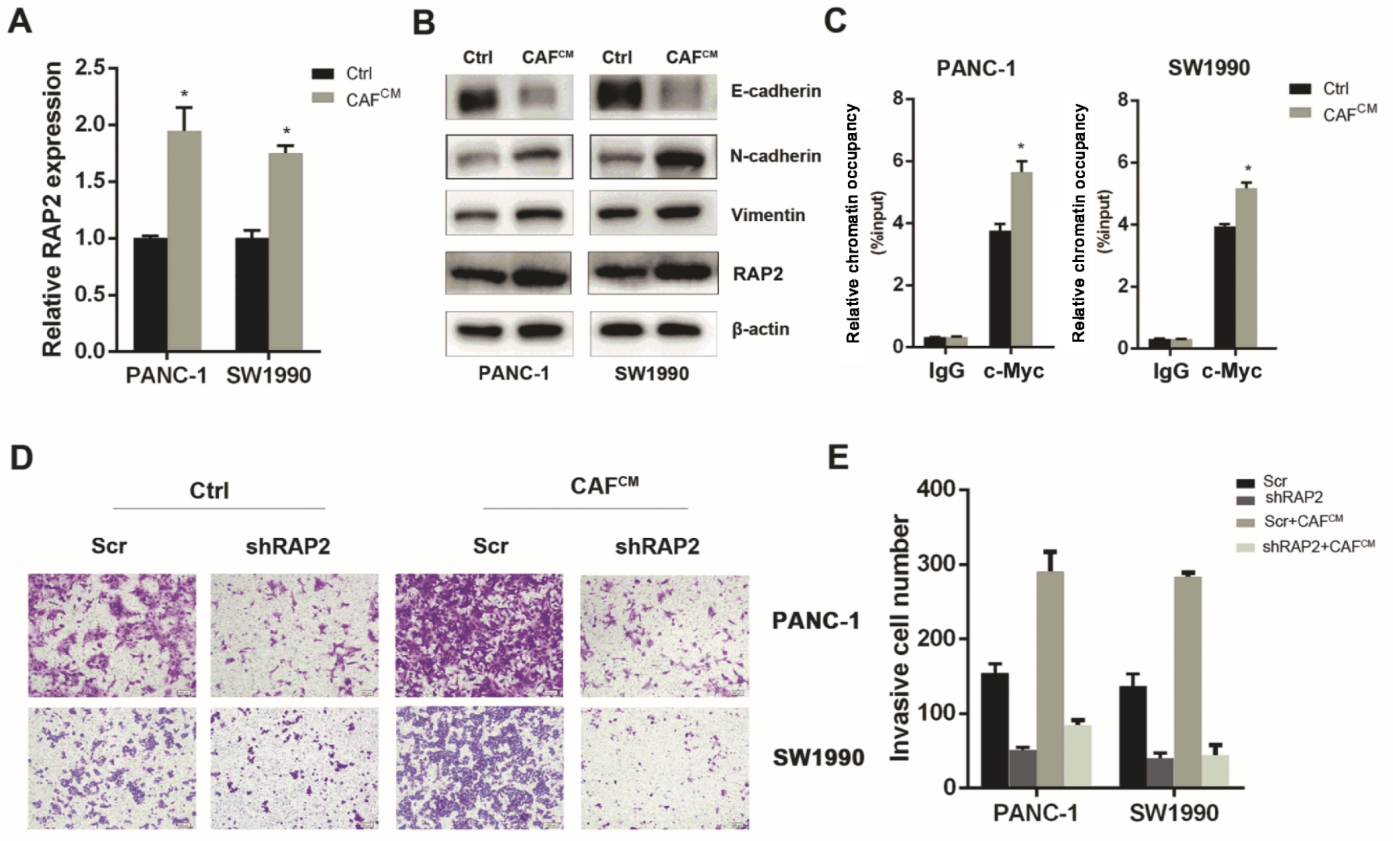
Figure5 CAFs induce RAP2 expression in pancreatic cancer cells (A) CAFCM treatment for 48 h induced RAP2 expression at mRNA level in PANC-1 and SW1990 cells. (B) Co-culture of PANC-1 and SW1990 cells with CAFCM increased the protein levels of RAP2, N-cadherin, Vimentin and decreased E-cadherin. (C) CAFCM increased the occupancy of c-Myc on RAP2 promoter region. (D) CAFCM induced migration and invasion capacity of PANC-1 and SW1990 cells (magnification, 100×). (E) Silencing of RAP2 attenuated the increase in invasion of PANC-1 and SW1990 cells.*P<0.05.

It is well accepted that CAFs can induce the invasiveness of cancer cells. To further confirm the above result under physiological conditions, we examined whether CAFs could increase the expression of RAP2 to induce the invasiveness of pancreatic cancer cells. First, we treated PANC-1 and SW1990 cells with CAF-conditioned medium (CAF ^CM^) for 48 h. Then, quantitative PCR assays were performed, and the results indicated that CAF ^CM^ induced RAP2 expression at the mRNA level in pancreatic cancer cells ( Figure 5A). By performing western blot analysis, we validated that co-culture of PANC-1 and SW1990 cells with CAF ^CM^ could increase the protein expression levels of RAP2, N-cadherin and Vimentin, while the protein level of E-cadherin was decreased ( Figure 5B). A subsequent ChIP assay revealed that CAF ^CM^ could increase the occupancy of c-Myc on the *RAP2* promoter region, which might be responsible for the induction of RAP2 expression ( Figure 5C). Finally, we asked whether RAP2 is responsible for CAFs-induced migration and invasion of pancreatic cancer cells. The results of Transwell invasion assays demonstrated that CAF ^CM^ induced the migration and invasion capacity of PANC-1 and SW1990 cells, but silencing of *RAP2* could attenuate the increase in invasion of PANC-1 and SW1990 cells ( Figure 5D,E). Collectively, these results demonstrated that CAFs induced migration and invasion in part through the c-Myc/RAP2 axis in pancreatic cancer cells.

## Discussion

**Figure 6:**
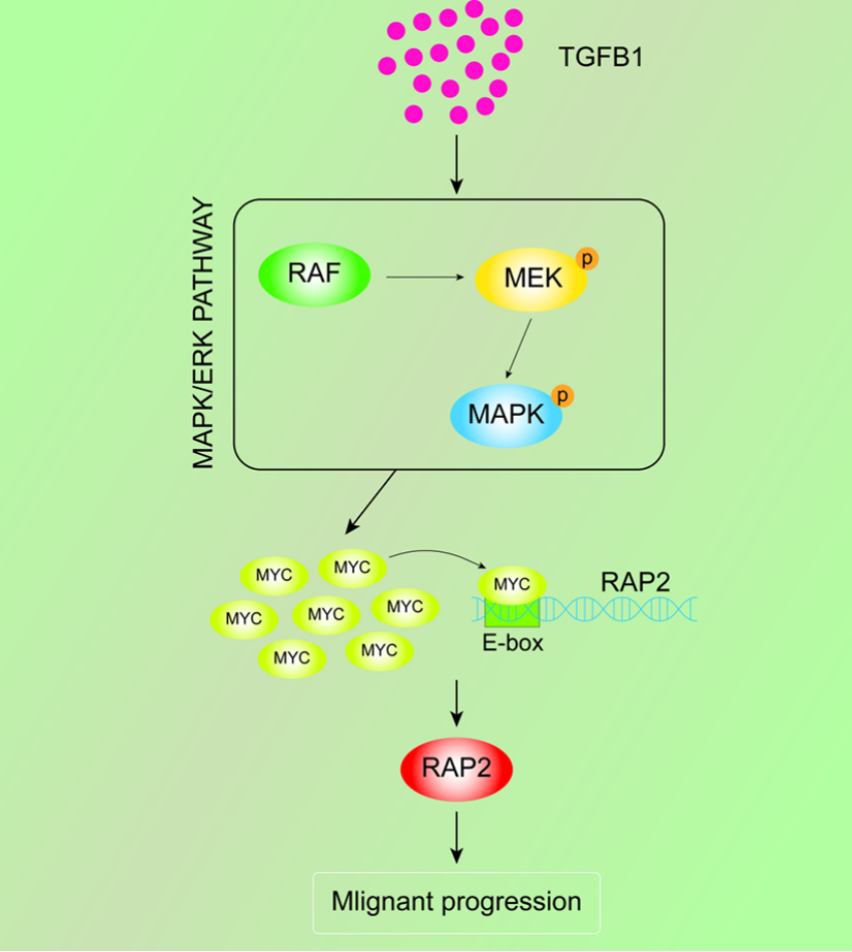
Figure6 Schematic overview of RAP2-mediated malignant progression in PDAC

Due to the high invasive and metastatic potential of pancreatic cancer, patients with this disease have a very poor prognosis, and attempts to target invasion for improving overall survival may provide a new window of opportunity [ 27, 28]. In the present study, we demonstrated that RAP2 could be utilized to predict overall survival. Mechanistic studies uncovered that RAP2 is induced by the TGF-β1/c-Myc axis and participates in the regulation of the invasiveness of pancreatic cancer cells ( Figure 6).

Consistent with our present results, many other studies have also demonstrated that RAP2 functions as an oncogene, and RAP2 is notably upregulated in human tumour samples and cancer cell lines, including prostate cancer [15], follicular thyroid cancer [29], lung cancer [30] and breast cancer [31]. RAP2 plays its tumour-promoting roles by many molecular mechanisms. For example, in osteosarcoma cells, RAP2 enhances cell migration and invasion via increasing MMP2 and MMP9 expression [32]. RAP2 also promotes renal cell carcinoma migration and invasion through up-regulating p-Akt [33]. In line with this observation, our present study uncovered that RAP2 might regulate the invasiveness of pancreatic cancer cells through its possible impact on the EMT process, as EMT-related metastatic markers changed significantly when RAP2 was silenced. However, there is a need for more evidence to validate the role of RAP2 in the EMT process. RAP2 might also regulate cancer cell malignancy via various signalling cascades. For example, TRAF2 and NCK Interacting Kinase (TNIK) is a serine/threonine kinase that functions in the activation of the Wnt signalling pathway [34]. RAP2 has been found to interact with TNIK through experiments using affinity purification-mass spectrometry [35]. In addition, RAP2 was reported to co-localize with TNIK when these two genes were co-expressed in cells [17]. TNIK can also interact with β-catenin and TCF4, leading to TCF4 phosphorylation and subsequent activation of Wnt target genes [36]. Therefore, RAP2 might regulate cancer cell proliferation and invasion via activating the TNIK/Wnt signalling cascade. Wnt signalling activation is also a driving force for pancreatic cancer oncogenesis, metastasis and progression [18]. Therefore, RAP2 might regulate the invasion of pancreatic cancer cells via the Wnt signalling pathway. Another possible signalling pathway that RAP2 might modulate during the regulation of the malignant properties of pancreatic cancer might be the activation of mitogen-activated protein kinase (MAP4K4). RAP2 interacts with MAP4K4 and enhances the MAP4K4-induced signalling pathway [37]. MAP4K4 is significantly upregulated in numerous cancer cell lines and tumors relative to their normal counterparts [ 38– 40]. MAP4K4 signalling regulates numerous cellular processes, including cell motility, cytoskeleton rearrangement, cell growth, cell migration and invasion [41]. Therefore, activation of the MAP4K4 pathway might be another reason for RAP2-induced migration of pancreatic cancer cells. Based on these data, there is a need to carry out further proteomic interaction screening and gene expression profiling to dissect the roles of RAP2 in pancreatic cancer oncogenesis, metastasis and progression.

Pancreatic cancer is characterized by an abundant desmoplastic tissue that accounts for nearly 80% of the total tumour mass [42]. This hallmark feature forms the intra-tumour microenvironment that consists of CAFs, immune cells and extracellular matrix (ECM) surrounding the cancer cells [42]. CAFs are the most abundant stromal cell type in pancreatic cancer [43]. Increasing evidence indicates that CAFs play vital roles in pancreatic cancer tumourigenesis, progression, metastasis, and drug resistance [44]. CAFs regulate the malignancy of pancreatic cancer cells via autocrine and paracrine signalling pathways [45]. In the present study, our results demonstrated that CAFs induced RAP2 expression via the transcription factor c-Myc. This is in line with the role of RAP2 in relaying cell/cell communication signals. c-Myc is the major driver of tumorigenesis in pancreatic cancer [46]. However, we did not further discuss whether TGF-β1 mediates the impact of CAFs on RAP2 expression in pancreatic cancer cells. In future studies, we will examine the level of TGF-β1 in CAFs medium. Moreover, to examine the impact of CAFs and TGF-β1 on RAP2 expression, neutralizing anti-TGF-β1 antibody or TGF-β1 cascade inhibitor SB431542 will be utilized to antagonize the TGF-β1 signal to block cell-cell interaction for uncovering whether the TGF-β1-c-Myc-RAP2 axis could mediate the interaction between CAFs and pancreatic cancer cells. Further investigations of the axis will help uncover novel strategies to block the interaction between CAFs and pancreatic cancer cells to reverse malignancies and metastasis of pancreatic cancer cells.

In conclusion, our present study demonstrated that RAP2 expression predicts worse outcomes of pancreatic cancer patients. *In vitro* cell line and mechanistic studies uncovered that RAP2 is positively regulated by c-Myc and induces the invasiveness of pancreatic cancer cells. Moreover, TGF-β1 activates c-Myc to induce expression of RAP2, leading to elevated invasion of pancreatic cancer cells. We further demonstrated that CAFs also induces c-Myc expression and occupancy on RAP2 gene promoter, leading to increased RAP2 expression. Collectively, the present study identified novel predictive markers and treatment targets for pancreatic cancer and pointed out directions for further investigations.

## Supplementary Data

Supplementary data is available at *Acta Biochimica et Biophysica Sinica* online.

## Supporting information

Supplementary_Table
