# Supplementary material for: TGF-β1-induced RAP2 regulates invasion in pancreatic cancer: RAP2 regulates invasion in pancreatic cancer
Source: Acta Biochim Biophys Sin (Shanghai). 2022 Feb 25;54(3):361–9. doi: 10.3724/abbs.2022015 (PMC9828032; doi:10.3724/abbs.2022015)
Supplement: Supplementary_Table [file Supplementary_Table.doc]

| **Supplementary Table S1. Characteristics of the patients** | | | |
| --- | --- | --- | --- |
|  | RAP2 Expression | |  |
| Variables | Low RAP2  (n= 35) | High RAP2  (n= 42) | *P* |
| Age |  |  | 0.967 |
| >60 y | 19 | 23 |  |
| ≤60 y | 16 | 19 |  |
| Sex |  |  | 0.834 |
| Male | 15 | 19 |  |
| Female | 20 | 23 |  |
| Location |  |  | 0.897 |
| Head | 13 | 15 |  |
| Body and tail | 22 | 27 |  |
| Primary Size |  |  | 0.880 |
| Φ≤4 cm | 27 | 33 |  |
| Φ>4 cm | 8 | 9 |  |
| Lymph metastasis |  |  | 0.577 |
| No | 23 | 25 |  |
| Yes | 12 | 17 |  |
| Vessel invasion |  |  | 0.820 |
| No | 25 | 29 |  |
| Yes | 10 | 13 |  |
| Perineural invasion |  |  | 0.131 |
| No | 6 | 2 |  |
| Yes | 29 | 40 |  |
| Differentiation |  |  | 0.191 |
| High/ Middle | 28 | 28 |  |
| Low | 7 | 14 |  |
| Cachexia |  |  | 0.206 |
| No | 32 | 33 |  |
| Yes | 3 | 9 |  |
| CA19-9 Level |  |  | 0.129 |
| ≤37 U/ml | 12 | 8 |  |
| > 37 U/ml | 23 | 34 |  |
